# Laparoscopic-assisted natural orifice specimen extraction surgery for colorectal cancer in patients with situs inversus totalis: a case series of four patients with outcomes (surgical video)

**DOI:** 10.1093/gastro/goag104

**Published:** 2026-10-15

**Authors:** Xiaoya Hou, Jixiang Liu, Qingpeng Meng, Yangyang Cao, Yipeng Deng, Junhong Hu

**Affiliations:** Department of Colorectal Surgery, the First Affiliated Hospital of Zhengzhou University, Zhengzhou, Henan, P. R. China; Department of Colorectal Surgery, the First Affiliated Hospital of Zhengzhou University, Zhengzhou, Henan, P. R. China; Department of Colorectal Surgery, the First Affiliated Hospital of Zhengzhou University, Zhengzhou, Henan, P. R. China; Department of Colorectal Surgery, the First Affiliated Hospital of Zhengzhou University, Zhengzhou, Henan, P. R. China; Department of General Surgery, Ward 2, Jiaomei Central Hospital, Jiaozuo, Henan, P. R. China; Department of Colorectal Surgery, the First Affiliated Hospital of Zhengzhou University, Zhengzhou, Henan, P. R. China

## Introduction

Situs inversus totalis (SIT) is a rare congenital anomaly characterized by mirror-image displacement of all thoracic and abdominal organs, with an incidence ranging from 1/8,000 to 1/25,000 [1]. When patients with SIT develop colorectal cancer, anatomical mirroring poses dual challenges to spatial orientation and surgical technique [2]. Natural orifice specimen extraction surgery (NOSES) offers advantages over conventional laparoscopy, including reduced postoperative pain, accelerated gastrointestinal recovery, improved cosmetic outcomes, and lower complication rates [3, 4]. However, reports of NOSES for patients with SIT with colorectal cancer remain rare [5]. This article presents a series of four patients with SIT and colorectal cancer who underwent laparoscopic NOSES, with complete surgical video (Supplementary Video S1) and oncological follow-up.

## Case report

From May 2022 to August 2024, our department admitted four patients with SIT complicated by colorectal cancer. Following multidisciplinary consultation, all patients underwent laparoscopic NOSES. Their clinical characteristics and surgical outcomes are summarized in Table 1. The most typical preoperative image is presented in Fig. 1.

**Figure 1:**
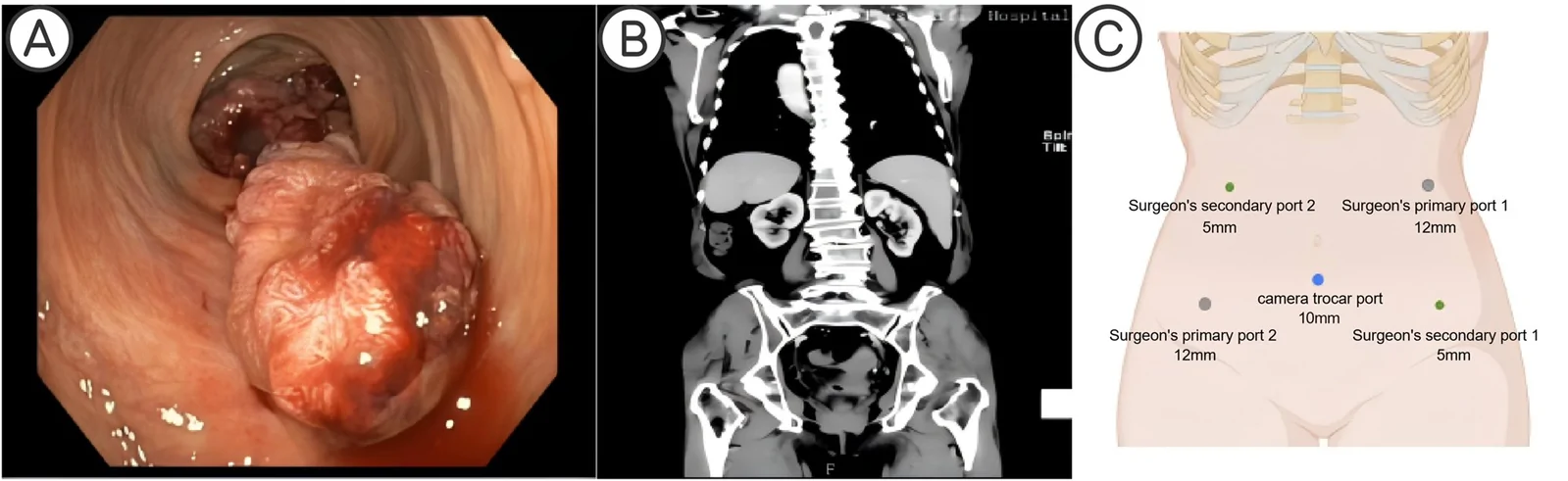
Preoperative imaging and port placement. (A) Case 1: Electronic colonoscopy revealed an irregular mass in the sigmoid colon (25 cm from the anal margin). Pathology revealed a villous tubular adenoma with focal malignant transformation. (B) Case 1: Enhanced CT of the chest and abdomen showing mirror-image dextrocardia with inversion of the abdominal viscera. A mass lesion in the sigmoid colon was suspected as cancerous. (C) Trocar placement diagram.

**Table 1:** Clinical characteristics and surgical outcomes of the four patients with situs inversus totalis and colorectal cancer.

Variable	Case 1	Case 2	Case 3	Case 4
**Sex/Age, years**	Female/75	Male/55	Male/68	Female/52
**Tumor location**	Sigmoid colon	Middle rectum	Upper rectum	Rectosigmoid junction
**Tumor size, cm**	4.7	3.2	3.6	2.8
**Operative time, min**	189	167	154	139
**Estimated blood loss, mL**	50	50	20	20
**Lymph nodes harvested, *n***	16	12	12	12
**Pathological type**	adenocarcinoma	adenocarcinoma	adenocarcinoma	adenocarcinoma
**pTNM staging**	pT3N0MX	pT3N1MX	pT2N0MX	pT2N0MX
**Adjuvant chemotherapy**	FOLFOX6	FOLFOX6	None	None
**Postoperative complication**	Intestinal obstruction^a^	None	None	None

a Resolved with conservative management.

## Surgical procedure

All procedures employed a five-port laparoscopic approach. A 10-mm trocar was placed at the upper edge of the umbilicus as the observation port. Two 12-mm trocars were inserted: one at the level of the umbilicus, just medial and superior to the left anterior superior iliac spine, and the other at the level of the umbilicus, at the lateral border of the right rectus abdominis muscle, serving as primary working ports. Two 5-mm trocars were inserted: one at the level of the umbilicus at the lateral border of the left rectus abdominis muscle, and the other in the right lower abdomen, serving as secondary working ports (Fig. 1C). A “dynamic primary surgeon station” strategy was adopted. The primary surgeon, initially positioned on the patient’s left side, managed the inferior mesenteric vessels and dissected the rectum distally to the pelvic inlet. The surgeon then shifted to the right side to complete total mesorectal excision (TME) and anastomosis. All specimens were retrieved transrectally without auxiliary abdominal incisions. No intraoperative vascular or neural injuries occurred. Median operative time was 160.5 minutes, and median blood loss was 35 mL. Postoperative pathological margins were negative. Follow-up was conducted via telephone and outpatient visits, with a median follow-up duration of 27 months (range, 16–43 months). No recurrence or metastasis was observed during this period.

## Discussion

With advances in minimally invasive surgical techniques, numerous investigations have been conducted on the surgical treatment of colorectal cancer in patients with SIT. However, most studies have focused on traditional laparoscopic or robot-assisted surgery, and many are case reports. In robotic surgery, numerous domestic and international scholars have confirmed that robotic systems, leveraging the advantages of 3D visualization, tremor filtering, and multiarticulated instruments, offer enhanced safety and convenience in anatomically complex procedures, and emphasize the importance of preoperative simulation and instrument arm mirroring settings [5–8]. All procedures reported in the literature were completed successfully, without any reported complications. In contrast to robotic approaches, our team reports for the first time four cases of successful laparoscopic NOSES procedures in patients with SIT and colorectal cancer. Among our patients, only one developed postoperative incomplete intestinal obstruction, which resolved with conservative management. No anastomotic leaks or other complications were observed, and postoperative oncological follow-up revealed no recurrence or metastasis. Our experience demonstrates that NOSES can be successfully performed through dynamic primary surgeon positioning, mirrored trocar placement, and team coordination, thereby eliminating the need for abdominal wall-assisted small incisions. Compared with traditional laparoscopic-assisted small-incision surgery, NOSES offers additional benefits for patients with SIT. It avoids the potential need for traditional auxiliary incisions for specimen extraction due to anatomical constraints, reduces incision-related complications (e.g. infection, hernia), and better meets patients’ aesthetic expectations regarding abdominal wall appearance. Although the median operative time in our cohort (160.5 minutes) was longer than the conventional NOSES duration reported in the literature [9], operative duration gradually decreased with increasing numbers of cases. This prolonged duration highlights the core challenge posed by SIT: the difficulty of reconstructing spatial cognition. Laparoscopic surgery relies on the surgeon’s deep “mental map” of normal anatomy. SIT fundamentally disrupts this mental map, requiring surgeons to continuously perform “mirror image conversion” during the procedure. Unconscious habitual maneuvers may increase the risk of inadvertent injury, particularly when handling critical structures such as the inferior mesenteric vessels and ureters. This cognitive load inevitably leads to more cautious manipulation, thereby prolonging the operative time to some extent. These findings further underscore the critical importance of meticulous preoperative planning and flexible intraoperative strategies. In summary, adjusting the surgeon’s position facilitates adaptation to the mirrored surgical field, whereas a rational trocar site layout significantly mitigates the negative impact of habitual left-right hand operating patterns.

## Conclusions

In conclusion, for carefully selected patients with SIT and colorectal cancer, laparoscopic NOSES using mirror-image trocar placement and dynamic primary surgeon positioning represents a safe and feasible minimally invasive treatment option. This approach fully embodies the modern surgical philosophy that, even under complex anatomical conditions, tumor radicality, functional preservation, and rapid recovery can be achieved through optimized surgical strategies rather than relying on expensive equipment.

## Supplementary Material

goag104_Supplementary_Data
